# The Anatomy, Physiology, and Diseases of the Bones

**Published:** 1835

**Authors:** 


### Art. IX.—Diseases of the Bones.

*The Anatomy, Physiology, and Diseases of the Bones. By Samuel D. Gross M. D., Professor in Cincinnati College. J. Grigg, Philadelphia,* 1830.

Books written upon the several branches of medical science have been so vastly multiplied, within the last quarter of a century, that one is sometimes ready to inquire, whether the state of our science still requires such continually accumulating additions to our libraries?

The medical reader who has kept pace with the advancement of the science, for the last half century, will readily admit that there has been, and continues to be ample room for pushing our inquiries vigorously, into every branch of medical science; but it is nevertheless usual and proper, that the medical inquirer should expect every author to acquaint us with the design, and particular end in view, in putting forth a new work.

In compliance with this usage Professor Gross has given such explanations as he deemed relevant to the publication before us. We are told in the preface that "excepting the lectures of Boyer, by M. Richerand and translated by Dr. Farrell, there is no treatise in our language in any degree commensurate with the subject before us. The works of Wilson, Brodie, Bell, Cooper, and others, although replete with information, are nothing but monographs, intended for the experienced practitioner rather than for the inexperienced student. In our own country, the diseases of the bones have hitherto received but little attention."

We believe that the foregoing assertion was not only correct at the time it was made, but that the condition of this matter has not been materially altered up to the present time—wherefore, we may readily acknowledge the utility of the work; and, we owe, to the author, more than our thanks for this successful effort to improve a very important subject.

"To supply deficiencies, and to furnish the student with a plain, concise and practical digest of the state of our science, embracing an account of the most important facts and observations, are the objects of the present undertaking."

We have been led, after a careful perusal, and mature reflection, on the work of Professor Gross, to aver, that he has well filled up the measure of his promise. The digest here presented is well calculated to introduce the inexperienced reader into sound elementary knowledge, in physiology, and pathology; nor will the experienced find a more ready book of reference, for what they may have forgotten, or may have escaped their notice.

Our author very modestly tells us that he makes no higher claim than that of a faithful compiler. In this we willingly offer our testimony to bear him out. The work contains a very considerable mass of highly useful matter, judiciously put together. Such being the character of the work, it will not be expected that we should undertake an analysis of its contents. We deem it sufficient in this place to state, that it exhibits, in general, correct principles, and the best established rules of practice. Whenever the author has advanced new or peculiar opinions, they are sufficiently pointed out.

We purpose, in turning over the pages before us, taking brief notices of what we may deem remarkable; and, candidly stating our views of such points as we may consider of sufficient interest, whether from novelty, defect of knowledge as generally understood, or belief of improvement.

At page 10, our author alludes to the fact, that the nerves are not to be traced into the substance, or interior of the bones, and, in the succeeding page, he states, as his opinion that "the nerves of the ossious tissue are conveyed to the bones through the medium of the arteries, that they enter the same foramena, and that they are, as it were, concealed in their coats, so as to escape our observation: this opinion, I find, is also entertained by J. C. Rosemuller, formerly professor of anatomy, in the univeisity of Leipzig."

There can scarcely be a doubt of the truth of the above opinion. We suppose every physiologist of the present day, will consider a nervous influence indispensable to the vitality of every organ, or tissue, of the animal body. It has been wisely contrived, that the parts which are subject to much mechanical attrition, either as supporters, or articular surfaces forming joints between the bones, as well as the tendons and ligaments, which are liable to violent extension, should be endowed with but little open or free sensibility; and hence it is, that nature has not supplied these tissues with nerves, in any degree proportioned to those distributed to parts, part of the natural function or office of which requires perceptibility in more or less force.

Now, it is well known, that some of the inferior animals; and the germinating rudiments of the more perfect animals, up to a certain period, exist and are developed with more or less rapidity, through a sensory having an interstitial placement co-extensive with the size, and having the form, of the entire animal body, whether in its various stages of growth, or after its completion. Then as the ulterior discovery, in the examination of animated beings has been to resolve them into molecules, we adopt the term molecular medulla as the name of a nervous matter, which is blended in the organization of every tissue, and in union with an influence pervading it at every point, exerted by the brain through the medium of the nerves.

We believe, that to this modification of the sensorial organs, associated with its share of the vivific principle, may be as cribed the fact of Haller's originating the opinion of a vis insita; and to it may we ascribe the plastic operations. I could not within the limits assigned this article, examine into this subject; we shall for the present content ourselves with a brief description of our views of the manner, in which the bones are associated with the more sensible parts or tissues.

We are not among those who ascribe the nervous energies to electrical influence, operating by any of its known laws, but we believe, that the nervous cords and fibres arise from the brain of the head and spine, and terminate in medullary substance, placed in every tissue; and, thus, in some degree, resembling the positive and negative electrical poles. The admission of such an arrangement will enable us to explain the manner by which the least sensible tissues hold their relative intercommunion of energy, life, health, or disease. Since pricking the skin with the point of the finest cambric needle, will cause severe pain, we may reasonably suppose, that no fibre is too small to give place to nervous influence—serving in the case before us, merely as conducting links, between the brain, as positive, and the medulla interstitially placed as negative points.

Now, we know that arteries are plentifully supplied with nerves, that these penetrate their coats, where the vessels and nerves are of sufficient size, to come within the power of our vision; can we then, for one moment doubt the assertion, that nerves having started, so to speak, along the arteries, will continue to the termination of the arteries, however minute they may become—indeed, to doubt under such circumstances the fact here advanced, can be nothing less than absurdity.

But it does not seem necessary that we insist upon the distribution of nervous fibres, through the more insensible structures—it is sufficient for our belief, of a capacity in these organs for assuming open sensibility, to know, that nervous fibres do at least reach the surface of such organs or tissues; and, may therefore, convey nervous influence, by means of these fibres, as conductors.

For ourselves, we have no doubt of the opinion, that nerves having set out to travel, so to speak, with the arteries, never end until they have reached the termination of the arteries, in all parts of the same individual; but, we think it highly probable, that a nervous influence exists between the uterus and the fœtus, by means of nervous points terminating on the inner surface of the uterus, and operating upon the nervous system of the fœtus, by an intervening molecular medulla existing in the placenta, and unbilical cord. There is strong hope, we think, that the views which we have presented above, will be ascertained to be correct, by experiment.—Professor Schlemm, of Berlin, has demonstrated the existence of nerves branching into, and through the cornea. The particulars of this discovery may be seen in Jameson's Medical Recorder, vol. 2, page 148.

Professor Gross, at page 16, speaks of the well known condition of the insensible tissues, which renders them liable not only to be thrown into the state of perceptibility, but of supersensation. Who, that has witnessed this state of things can doubt the existence of a sensorial material in those tissues. But as we have already said, nature has wisely suited the inherent sensory of these structures, to their exposed condition; instead of degrading them to a state almost inanimate, as has too often been imagined.

It would seem more consistent, with our present views of physiology, to distinguish sensibility into different modifications; and, we shall find, that on the one hand, nature designed that perceptibility should exist, as a condition through which organs or tissues were to perform their offices; so, on the other hand, where it was evident that perceptibility would go to unfit parts exposed to strong mechanical forces for their offices, they were made to possess a diminished amount of it.

Parts subjected to volition are endowed with a high state of sensibility, this may be denominated free or open sensibility. Parts not subject to volition have impressed upon them a capacity to take on sensibility when invaded by disease, this may be termed sub-sensibility.

Page 36 it is said--"Dr. Physick the distinguished Professor of Anatomy in the University of Pennsylvania, is of opinion that the periosteum often prevents the bones from participating in the diseases of the neighboring parts, in the same manner "as the pleura turns off an abscess in the parietes of the thorax from its cavity, or the peritoneum from the cavity of the abdomen."—(*Wistar's Anatomy.*)

We are aware of the truth of the fact here pointed out, —but it by no means applies specially to the periosteum, and pleura, it was well known to Mr. John Hunter, as a general law; and served to form one of his distinctions of sympathy. Simple abscesses do not materially involve the blood vessel, over, or near, which they are situated; inflammation, of the scrotum from injections into the cyst of the hydrocele seldom extends to the testicle; inflammation of the anus never extends from the skin to the rectum—suppurations in the perineum will expose the urethra for two or three inches, and leave it almost free from the effects of the suppuration, etc., etc.

This compendium contains *two parts—Part 1st, is arranged in three chapters.* The first chapter treats on the anatomy and physiology of the bones, and their membranes.

In this chapter, of upwards of thirty pages, we have presented a judicious selection of what is known on the subjects therein treated; and, we hazard nothing in saying, that no work can be found in which a greater amount of correct information can be seen within the same limits—the arrangement and matter are good, and presented in language well suited, being alike brief and lucid.

The second chapter treats on fractures or injuries of the bones. Section 1st of fractures in general. 2d—of particular fractures.

Chapter third, treats upon diseases of the bones. Section 1st, of caries—2d, of necrosis—3d of osteo sarcoma—4th, of spina ventosa—5th, of osteo hydatical tumors—6th, of rachitis—7th, of hydro-rachitis—8th, of mollities ossium—9th, of fragilitas ossium—10th, of periostosis—11th, of exostosis—12th, osteo-sanguineous tumors—13th, insterstitial absorption of the cervix femoris.

The three foregoing chapters contain a considerable collection of information highly interesting, since it embraces views of the injuries, and diseases treated of, as well as modes of treatment, embracing whatever is well established, by the most experienced writers, on the several subjects embraced within these chapters.

*Part second* is also arranged in three chapters. Chapter 1st, treats on the general anatomy of the joints. Sections 1st, 2d, 3d, and 4th, treat on the anatomy of the cartilages membranes, articular fluids, and ligaments.

Chapter 2d, of injuries of the joints. Section 1st, of dislocations in general—2d, of particular dislocations—3d, of wounds of the joints—4th of sprains.

Chapter 3d, of diseases of the joints. Section 1st, of inflammation of the synovial membranes—2d, hydrarthrus—3d, of inter-articular cartilaginous concretions—4th, of fungus articuli—5th, of fermero-coxalgia—6th, loxarthrus—7th, of anchylosis, etc.

The mere inspection of the above catalogue of injuries, and other diseased states of the bones, membranes, joints, etc., is sufficient to satisfy every one, that if these various affections and injuries, are properly managed, in regard to their characters and modes of treatment, it follows, as a matter of course, that the work before us contains a very considerable amount of information. I know of nothing of equal extent, that can be obtained from any quarter whatsoever, containing more practical matter than the compendium here offered of the physiology,and pathology of the bones etc., that it contains a judicious selection of what is most valuable, I most willingly state as my opinion. I shall be pleased to learn that merit may find a reasonable and just reward in the free discrimination of this truly useful work. H. G. J.

